# 886. Navigating Ethical Challenges of a Family Burned: A Child Life Specialist's Perspective

**DOI:** 10.1093/jbcr/irag033.372

**Published:** 2026-04-06

**Authors:** Emily Rosenbaum, Marcie A Lambrix, Casey L Kohler, Hannah Martin

**Affiliations:** MetroHealth Hospital, Broadview Heights, OH; The MetroHealth System/Case Western Reserve University, Cleveland, OH; MetroHealth Hospital, Cleveland, OH; MetroHealth Hospital, Cleveland, OH

## Abstract

Ethical issues in burn care continue to gain increased attention among the burn community (Gerrek, 2018; Teven & Gottlieb, 2018). This paper adds to the current burn ethics literature by highlighting the unique ethical challenges an urban, Midwestern ABA verified burn unit faced after a massive explosion struck an urban multi-building apartment complex. The explosion resulted in an apartment fire with three of the four family members, including two children and their stepdad, being critically burned and admitted to the burn unit.

This case study employs a narrative approach to unpack the ethical challenges that emerged while caring for both school aged kids, both of whom sustained roughly 50% TBSA injuries, and their stepdad, who sustained an alarming 90% TBSA injury. While many challenges encompassed the family unit throughout admission, particular emphasis here highlights the ethical challenges from the perspective of the BICU Child Life Specialist.

Ethical issues that emerged while caring for this family included maintaining privacy and confidentiality given the burn units' small size and patient’s close proximity to one another, making it difficult to keep patient care and medical updates private. Additionally, providers struggled with balancing being honest with the kids about one another’s prognosis, while also trying to protect them from the psychological distress that could impact their recovery. This delicate balancing of truth telling, beneficence and non-maleficence was particularly challenging given that once both kids were extubated, they recalled their stepdad going back into the apartment to “save them.” While the kids worked through their trauma, acute stress disorder, and ACEs, they attempted to cope with the ambiguous loss of their home and former lives. However, the ultimate loss came when stepdad died, 3 weeks post-explosion.

Caring for burn patients presents many ethical challenges, but caring for a family of burn patients is even more demanding. The burn team was challenged with how to navigate advocating for the pediatric patient’s emotional safety and psychosocial wellbeing while caring for their burn injuries. We emphasize the importance of Child Life Specialists in helping to navigate these ethical issues, including truth telling, beneficence, and justice more broadly.

